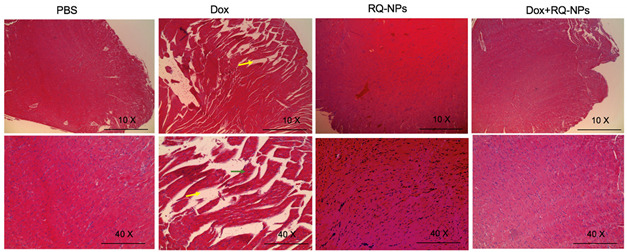# Correction: Godugu et al. Nanoformulated Ajwa (Phoenix Dactylifera) Bioactive Compounds Improve the Safety of Doxorubicin without Compromising Its Anticancer Efficacy in Breast Cancer. *Molecules* 2020, *25*, 2597

**DOI:** 10.3390/molecules27248730

**Published:** 2022-12-09

**Authors:** Kavitha Godugu, Ali H. El-Far, Soad Al Jaouni, Shaker A. Mousa

**Affiliations:** 1 Pharmaceutical Research Institute, Albany College of Pharmacy and Health Sciences, Rensselaer, NY 12144, USA; 2 Department of Biochemistry, Faculty of Veterinary Medicine, Damanhour University, Damanhour 22511, Egypt; 3 Department of Hematology/Pediatric Oncology, Yousef Abdulatif Jameel Scientific Chair of Prophetic Medicine Application, Faculty of Medicine, King Abdulaziz University, Jeddah 21589, Saudi Arabia

## Error in Figure

In the original publication [1], there was an error in the representative images for PBS (control) and RQ-NPs (10× and 40×), which were mistakenly duplicated while arranging the figure from the control and the RQ-NPs sets of the cardiac tissue slice images. Corrected representative images at 10× and 40× are placed from the RQ-NP treated group cardiac tissue slices. The authors apologize for this unintentional mistake. These changes in the representative images do not affect the conclusions regarding the biological activity of the RQ-NP with or without Dox studied as shown in Figure 6.

Corrected Figure 6: